# Reimagining Care: Technology-Enabled Strategies To Address The Direct Care Workforce Shortage

**DOI:** 10.1093/geroni/igaf122.4401

**Published:** 2025-12-31

**Authors:** Sharmila Suresh, Jara Pallas-Brink

**Affiliations:** Michigan State University, East Lansing, Michigan, United States; Michigan State University, East Lansing, Michigan, United States

## Abstract

Direct Care Workers (DCWs) provide long-term care and personal assistance services to older adults and those living with disabilities or chronic conditions, ensuring a high quality of life and independence. The shortage of Direct Care Workers in the United States has become a critical challenge for aging and disability services. Contributing factors are high turnover rates, lack of benefits, low wages, racism, lack of respect, and inadequate training. To address the DCW shortage, IMPART Alliance at Michigan State University launched a program that supports employers in testing innovative retention and recruitment strategies. Within this context, this paper examines several organizations that are piloting grassroots innovations that integrate technology into care models. One promising example is to expand residential supported housing offerings by introducing a smart home alternative to traditional residential support models which rely on 24-hour direct support. This initiative aims not to replace DCWs but to utilize technology as a tool to elevate person-centered care, expanding opportunities for communication and enhancing individualized support. Furthermore, these programs emphasize training and the development of new skills, fostering a collaborative relationship between caregivers, technology, and clients. By examining three organizational initiatives that focus on various innovations in care, this paper explores the potential for grassroots, technology-enabled solutions to both strengthen the direct care workforce and inform broader strategies for addressing the shortages.

